# Technique of pressurized intrathoracic aerosol chemotherapy (PITAC) for malignant pleural effusion

**DOI:** 10.1515/pp-2020-0129

**Published:** 2020-11-09

**Authors:** Gabrielle Drevet, Jean-Michel Maury, Naoual Bakrin, François Tronc

**Affiliations:** Department of Thoracic Surgery, Lung and Heart-Lung Transplantation, Louis Pradel Hospital, Hospices Civils de Lyon, Lyon, France; Viral Infection and Comparative Pathology (IVPC), UMR 754, Claude Bernard Lyon 1 University, Lyon, France; Department of General Surgery and Surgical Oncology, Centre Hospitalier Lyon-Sud, Hospices Civils de Lyon, Pierre-Bénite, France; EMR 3738 Lyon Sud Charles Mérieux Faculty, Claude Bernard University Lyon 1, Oullins, France

**Keywords:** malignant pleural effusion, PITAC, pleural metastasis, Pressurized IntraThoracic Aerosol Chemotherapy

## Abstract

**Objectives:**

Malignant pleural effusion (MPE) is a devastating evolution of several malignancies. Pressurized intrathoracic aerosol chemotherapy (PITAC) might be a novel therapy option in MPE.

**Methods:**

PITAC is considered for patients with MPE with a performance status <2 and without other metastatic sites. General anesthesia is administered and a double-lumen bronchial tube is inserted. The patient is placed in a lateral decubitus position, and the operation is performed after ipsilateral lung exclusion. Two 12-mm balloon trocars are inserted—one in the seventh intercostal space in the mid-axillary line and one in the fifth intercostal space in the anterior axillary line. Extent of pleural disease and volume of MPE are documented. MPE is removed and parietal pleural biopsy are performed. An intrathoracic pressure of 12 mmHg CO_2_ is established, and a combination of Cisplatin (10.5 mg/m^2^ in a total volume of 150 cc NaCl 0.9%) and Doxorubicin (2.1 mg/m^2^ in a total volume of 50 cc NaCl 0.9%) are aerosolized via nebulizer in the pleural cavity. Vital signs and nebulization are remote-controlled. After 30 min, the remaining toxic aerosol is exhausted using a closed surgical smoke evacuation system. A 24Fr chest tube is inserted in postero-apical position with continuous negative pressure of 20 cm H_2_O. When needed, PITAC may be repeated every six weeks in alternate with systemic chemotherapy.

**Results:**

In our hands, the technique above has shown to be feasible and safe.

**Conclusions:**

Further studies are needed to assess the potential symptomatic and oncological benefits of PITAC in MPE.

## Introduction

Malignant pleural effusion (MPE) is a typical evolution of various cancers associated with poor prognosis and reduced quality of life. Median survival ranges from 3 to 13 months, depending on the primary malignancy [1]. The current therapeutic approach is mainly palliative, involving videothoracoscopic (VATS) talc pleurodesis or indwelling pleural catheters insertion. Depending on the patient’s general condition, local measures are associated with systemic chemotherapy.

A similar situation is observed in patients with peritoneal metastasis (PM). The relative lack of efficacy of systemic therapy led to the development of locoregional approaches such as hyperthermic intraperitoneal chemotherapy (HIPEC) and, in unresectable PM, pressurized intraperitoneal aerosol chemotherapy (PIPAC). PIPAC can induce objective histological regression of PM in end-stage, therapy-resistant patients, preserves their quality of life, and provides effective ascites control [2]. Given the promising results of PIPAC in PM, we applied this innovative technique for the pleura. Here we describe our technique for pressurized intrathoracic aerosol chemotherapy (PITAC) in patients with MPE.

## Materials and methods

### Indication

PITAC procedure is considered for patients with malignant pleural effusion proved by pleural fluid cytology, with a performance status <2 and without any other metastatic site. Due to the lack of high-level evidence, the indication for a PITAC procedure is an individual decision. Before therapy, all patients who could benefit from PITAC should be presented to the multidisciplinary tumor board. The combination with systemic chemotherapy should be decided on a case-by-case basis. Patients receive extended, detailed oral and written information about all available treatment options as well as the nature and risks of PITAC as a novel procedure involving the off-label use of approved chemotherapeutic drugs. Each patient is requested to provide written informed consent prior to surgery.

### Technique

**Figure 1:**
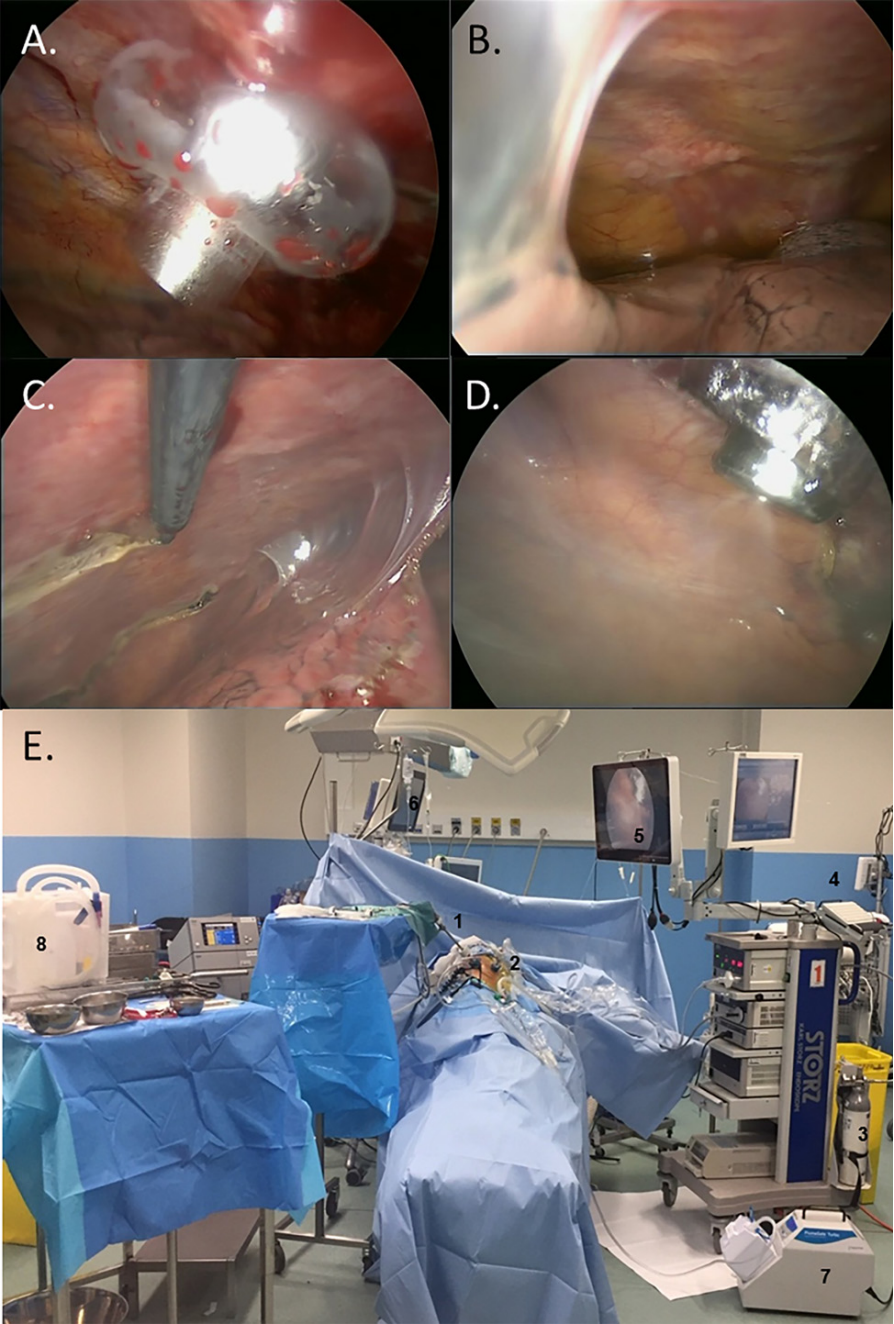
Peroperative intrathoracic views. (A) 12-mm balloon trocar insertion. (B) Pleural cavity exploration showing diffuse pleural nodules, multiple pleuro-pulmonary adhesions, and moderate pleural effusion. (C) Representative pleural biopsy. (D) Real-time endoscopic monitoring of the nebulization, controlling the correct position of the nebulizer and the absence of contact with the lung. (E) Operating room during nebulization. The patient is left alone with remote monitoring of vital signs and nebulization on respective screens. 1. Videomonitoring. 2. Nebulizer. 3. CO_2_ insufflator. 4. High pressure chemotherapy injector. 5. Screen monitoring the nebulization. 6. Screen monitoring the vital signs. 7. Closed aerosol evacuation system. 8. Material prepared for chest tube insertion and wound closure after aerosol evacuation.

Similarly to most VATS procedures, general anesthesia is administered and a double-lumen bronchial tube is inserted. A dedicated checklist, containing all safety aspects of the PITAC procedure [3], [4], is double-checked before administration of cytostatics. The patient is placed in a lateral decubitus position, and the operation is performed after ipsilateral lung exclusion. Two 12-mm balloon trocars (Applied Medical, Düsseldorf, Germany) are inserted into the chest wall: one in the seventh intercostal space in the mid-axillary line and the second one in the fifth intercostal space in the anterior axillary line (Figure 1A). The extent of metastatic pleural involvement is documented and the volume of the MPE quantified (Figure 1B). After removal of the effusion, parietal pleural biopsies are taken (Figure 1C). These biopsies are needed to confirm the metastatic nature of the pleural deposits and will serve to assess tumor histological response if further PITAC procedures are required.

**Figure 2:**
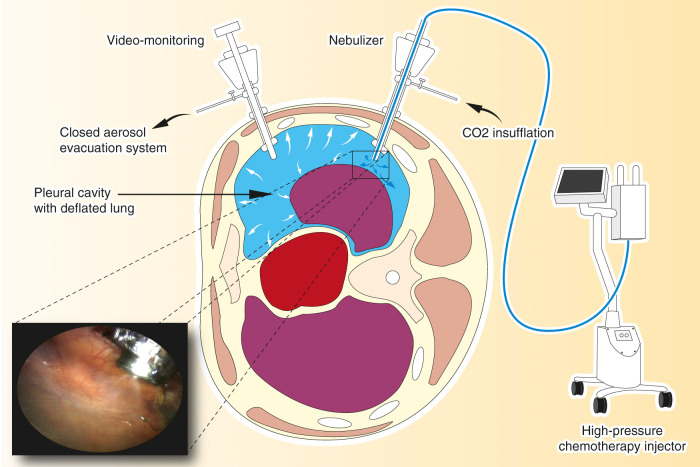
Pressurized intrathoracic chemotherapy (PITAC). The procedure is performed in an operating room equipped with an advanced air filtering system. A balloon trocar is inserted and the camera is introduced to explore the pleural cavity. A second balloon trocar is inserted, permitting pleural effusion removal, pleural biopsies, and finally, the placement of the nebulizer. An intra-thoracic pressure of 12 mmHg CO_2_ is established, and the chemotherapy is aerosolized over a period of 6 min. The system is maintained in steady-state for 30 min. At the end of the procedure, the toxic aerosol is exhausted through a closed aerosol evacuation system (CAWS).

An intra-thoracic pressure of 12 mmHg CO_2_ is established, and a dedicated, CE-certified nebulizer (Capnopen^®^, Capnomed, Zimmern o.R., Germany) is inserted through a trocar and connected to a high-pressure injector (Accutron HP-D, Medtron, Saarbrücken, Germany). All the staff leave the operating room to prevent exposure to chemotherapy (Figure 1E). Using a remote-control, Cisplatin (10.5 mg/m^2^ in a total volume of 150 mL NaCl 0.9%) and Doxorubicin (2.1 mg/m^2^ in a total volume of 50 mL NaCl 0.9%) are aerosolized into the pleural cavity at a flow of 0.5 mL/s (Figure 1D). Vital signs and nebulization are remote-controlled by the surgeon and the anesthesiologist from outside the operating room, in our hospital through a glass window. Since the air in the operating room is rapidly cleaned with high-performance (HEPA) filters, it is always possible to enter the operating room with no significant inhalation risk [3]. The system is left in steady-state for 30 min at a constant pressure of 12 mmHg to increase drug penetration into the neoplastic tissue. After this period, the staff enters the operating room wearing aerosol masks, and the remaining toxic aerosol is exhausted using a closed surgical smoke evacuation system (PlumeSafe Turbo, Conmed, Utica, NY) (Figure 2). A 24Fr chest tube is inserted in postero-apical position and connected to a Pleur-evac system (Teleflex, Morrisville, NC) with continuous negative pressure of 20 cm H_2_O.

Every month postoperatively, dyspnea and general condition are assessed. A chest X-ray is scheduled to detect pleural effusion recurrence. PITAC may be repeated every six weeks, possibly alternating with systemic chemotherapy in case of recurrence of a significant pleural effusion.

## Discussion

Here in this article, we describe our indications and technique for performing PITAC in patients diagnosed with MPE. In our hands, we have found PITAC to be feasible and safe. The patient selected for the first PITAC procedure in our institution was a 72 year-old man with a medical history of gastric adenocarcinoma. Eight months after gastrectomy, the patient developed MPE. A collegial discussion led to the decision of PITAC procedures alternating with systemic chemotherapy. The postoperative course was uneventful, and three months postoperatively, pleural effusion had not reappeared. Given the absence of recurrent pleural effusion, further PITAC procedures were not required.

Currently, the therapeutic approach of pleural metastasis is not specific enough, and the prognosis of this disease remains poor. Hyperthermic intrathoracic chemotherapy (HITHOC) combined with pleurectomy has shown to be a safe option and provided prolonged disease-free and overall survival in selected patients with pleural recurrence of thymoma [5]. Cytoreductive surgery combined with HITHOC was also performed in selected patients diagnosed with malignant pleural mesothelioma with acceptable morbidity and mortality rates [6]. In this indication, results of multimodality therapy (cytoreductive surgery combined with HITHOC, perioperative chemotherapy, and adjuvant hemithoracic radiation-therapy) on median survival are encouraging [7]. These promising outcomes should encourage the development of innovative approaches in the management of patients with advanced pleural metastasis, and surgical options in MPE should now include PITAC. By analogy with peritoneal diseases, the prognosis of patients with MPE could significantly improve.

However, the evidence published on PITAC is minimal. The first PITAC was performed by M.A. Reymond in Herne, Germany in April 2012 [8]. Between April 2012 and April 2014, the Herne group applied 10 PITAC in six patients for MPE of gastric origin (*n*=4), ovarian origin (*n*=1) or malignant mesothelioma (*n*=1). In four instances, (one-sided) PITAC was combined with PIPAC. The patient with malignant peritoneal and pleural mesothelioma had three PITAC, one on the left side, two on the right side, as well as four PIPAC, combined in four procedures in six-week intervals over six months. Operating time for PITAC alone was 100 ± 25 min, operating time for combined PITAC and PIPAC procedure was 189 ± 29 min. No intraoperative complication was noted, and the procedure was well tolerated in all patients. Hospital mortality was zero. No postoperative complication CTCAE>2 was noted. Control of the pleural effusion was achieved in all cases.

Later on, the same group published five PITAC procedures in three patients with malignant mesothelioma; all of them underwent simultaneous PIPAC procedure [9]. No postoperative complications occurred. PITAC was repeated at an interval of six weeks (in accordance with PIPAC protocols) for the recurrence of a significant MPE in two patients. Follow-up CT scans showed stable conditions. Further PITAC procedure was finally not performed in one patient because of a rapid disease progression and decline of general condition. Another patient to benefit from PITAC was diagnosed with a pleural extension of Pseudomyxoma Peritonei [10], and the postoperative course was uneventful. The most extensive series of PITAC reported on 21 PITAC in 10 patients, four receiving only PITAC, six combined PITAC and PIPAC procedures. Access to the chest was not a problem in any case. There was no intraoperative complication during PITAC. The hospital stay was almost uneventful and did not influence the outcome of PITAC [11].

In conclusion, our experience in hyperthermic intrathoracic chemotherapy [12] and the promising results of PIPAC [13] led us to introduce PITAC procedures in our institution. We provide a detailed description of this technique that could be useful to standardize practices and subsequently enable large-scale studies. At this stage, PITAC feasibility has been demonstrated, but its efficacy is not known yet. A randomized controlled study will be needed to assess the benefit of PITAC in improving the prognosis of patients with MPE.
